# Research on a Pedestrian Crossing Intention Recognition Model Based on Natural Observation Data

**DOI:** 10.3390/s20061776

**Published:** 2020-03-23

**Authors:** Hongjia Zhang, Yanjuan Liu, Chang Wang, Rui Fu, Qinyu Sun, Zhen Li

**Affiliations:** School of Automobile, Chang’an University, Xi’an 710064, China; zhanghongjia@chd.edu.cn (H.Z.); 2018122044@chd.edu.cn (Y.L.); furui@chd.edu.cn (R.F.); sunqinyu@chd.edu.cn (Q.S.); lizhen@chd.edu.cn (Z.L.)

**Keywords:** natural observation data, pedestrian intention recognition, fully automated vehicle, intention parameter set, attention mechanism

## Abstract

Accurate identification of pedestrian crossing intention is of great significance to the safe and efficient driving of future fully automated vehicles in the city. This paper focuses on pedestrian intention recognition on the basis of pedestrian detection and tracking. A large number of natural crossing sequence data of pedestrians and vehicles are first collected by a laser scanner and HD camera, then 1980 effective crossing samples of pedestrians are selected. Influencing parameter sets of pedestrian crossing intention are then obtained through statistical analysis. Finally, long short-term memory network with attention mechanism (AT-LSTM) model is proposed. Compared with the support vector machine (SVM) model, results show that when the pedestrian crossing intention is recognized 0 s prior to crossing, the recognition accuracy of the AT-LSTM model for pedestrian crossing intention is 96.15%, which is 6.07% higher than that of SVM model; when the pedestrian crossing intention is recognized 0.6 s prior, the recognition accuracy of AT-LSTM model is 90.68%, which is 4.85% higher than that of the SVM model. The determination of pedestrian crossing intention parameter set and the more accurate recognition of pedestrian intention provided in this work provide a foundation for future fully automated driving vehicles.

## 1. Introduction

### Background

According to traffic accident statistics released by the Chinese government, 63,093 people died in traffic accidents in China in 2016. Among them, the number of pedestrian deaths caused by vehicle impacts was 16,525, accounting for 26.2% of all deaths, while the number of people injured was 40,114, accounting for 17.7% of all injuries. Data from China’s road safety administration on pedestrian accidents related to zebra crossings shows that from 2015 to 2017 there were 14,000 vehicle-pedestrian collisions on zebra crossings in China, resulting in 3898 deaths [1].

The number of accidents at zebra crossings can be lowered significantly if vehicles are able to understand pedestrian intentions before crossing. Due to rapid developments in technology, a fully automatic driving system will be available in the very near future. Such vehicles have significant potential to reduce collision-related casualties while improving traffic conditions and reducing traffic congestion and vehicle emissions. In 2017, the United States Department of Transportation issued Automated Driving Systems 2.0: A Vision for Safety [2], aiming to improve the safety and reliability of the automatic driving system to reduce accident rates. Previously, in 2016, the Society of Automotive Engineers of China issued an automated driving technology roadmap [3], in which it was noted that every vehicle should be equipped with automated driving or auxiliary driving systems by 2026–2030. It is therefore evident that the autopilot system is considered highly important all over the world.

Many challenges remain in the development of automated driving technology. Aside from issues associated with developing suitable infrastructure [4] and regulating autonomous cars, technologies currently used in autonomous vehicles have not achieved the level of robustness to handle various traffic scenarios such as varied weather, lighting conditions, road types or environments [5]. In addition, for vehicles driving in a more complex traffic scene, especially in the urban environment, autonomous vehicles also face the additional challenge of how to achieve effective interaction with other road users. This task provides the key to reducing the accident rate by accurately identifying the intention of road users and then making the most correct and reasonable decision. Failure to effectively identify the intention of travelers will lead to traffic accidents, such as those experienced by Google’s autonomous vehicle.

Understanding pedestrian crossing intention in the unsignalized road section is one of the most important tasks for autonomous vehicles at present. By accurately identifying the pedestrian crossing intention in front of the vehicle, the system can engage vehicle deceleration in advance, avoid collision with the pedestrian, and improve safety. Additionally, if the vehicle can accurately judge there is no pedestrian crossing intention, it can directly drive at the original speed, improving driving comfort and efficiency. At present, most existing research related to intention recognition is divided into two categories: human posture language-based and pedestrian motion estimation-based.

- Human posture language:

Raul et al. [6] proposed a method to predict the future trajectory, posture, and intention of pedestrians. By balancing the Gaussian process dynamics model (B-GPDMs), the key points or joints of pedestrians are extracted, then the trajectory and intention of pedestrians is inferred. Recognition accuracy using this method reaches up to 80%. Raul et al. [7,8] also presented a method of pedestrian intention recognition based on a hidden Markov model. The method employs 11 key three-dimensional positions and displacements on the human body to identify pedestrians, reaching an accuracy of 80% intention recognition 125 ms after the start of the movement. Fang et al. [9,10] continuously extracted the feature vectors of T frame images and input them into SVM to identify pedestrian starting, crossing, and stopping intentions by locating human body key points and calculating the angle, distance, and other feature vectors between key point, attaining 93% accuracy of intention recognition. Flohr et al. [11] also proposed a method to estimate pedestrian orientation based on head and body orientation approximations, reaching an accuracy of 90%. Variimidis et al. [12] focus on the motion and head orientation to predict whether the pedestrian is about to cross the street or not. An accuracy of 72% for head orientation estimation and 85% for motion detection is obtained. Rasouli et al. [5,13] used AlexNet to extract features related to pedestrian movement and the surrounding environment, extracted t frames continuously to construct feature matrix, and input this into linear SVM to determine whether pedestrians will cross the street. Ghori et al. [14] proposed a real-time learning framework based on the relationship between human posture and intention to realize pedestrian intention recognition. The results show that this method can detect the intention earlier and more definitely and has the ability to recognize the intention of the traveler 1 s in advance. Schulz et al. [15] and Brouwer et al. [16] identified whether pedestrians had the intention to cross the street after estimating the head posture of the pedestrian. These vision-based intention estimation algorithms often treat the problem as dynamic object tracking by taking into account changes in the position, velocity, and orientation of pedestrians [17]. Köhler et al. [18] focuses on monocular-video-based detection of the pedestrian’s intention. on average it allows for detection of the movement within 6 frames at a frame rate of 50 Hz and an accuracy of 80 %.

- Pedestrian motion estimation:

Völz et al. [19] predicted pedestrian intention by the pedestrian’s movement track. The prediction results show that using a very sparse feature set, the prediction results are better, with prediction accuracy reaching 91.67%. In another study, Völz et al. [20] used long-term and short-term memory networks to predict the intention of pedestrians, collecting pedestrian crossing data through laser scanner and obtaining model input after preprocessing the collected two-dimensional point cloud image. Using this method, after verification, the recognition accuracy of the recognition results is improved by nearly 10–20% compared with that of the SVM algorithm. In [21], Völz et al. proposed a pedestrian intention prediction method combining the pedestrian motion tracking algorithm and data-driven method, which improved the generalization ability of the model. In references [19,20,21], the input variables of the model proposed are not considered comprehensively, and the model may not reflect pedestrian intention accurately. Camara et al. [22] proposed an intention heuristic model using input parameters including pedestrian trajectory, vehicle trajectory, and relative position. On the Daimler standard pedestrian data set, the crossing intention of pedestrians when they arrive at the roadside is predicted with 96% accuracy. Zhao et al. [23] put forward an improved naive Bayesian method, which can effectively identify pedestrian intention 0.5 s before the pedestrian crossing. Hashimoto et al. [24] proposed a dynamic Bayesian network model of the relationship between the intersection environmental information and pedestrian behavior. The results show that the model can identify the pedestrian crossing decision from the pedestrian location information. Schneemann et al. [25] further improved detection by presenting a context-based pedestrian motion history image and SVM model for pedestrian intention recognition. Hashimoto et al. [26] considered contextual information about the scene such as signal status, whether pedestrians are walking alone or in groups, and how close they are to the curb to identify intentions. Skovierov et al. [27] collected the position, speed, and orientation information of all traffic participants of pedestrians, and realized the recognition of pedestrian intention through the Bayesian network. Zhao et al. [28] collected the speed, position, and direction data of pedestrians through lidar, and used deep autoencoder–artificial neural network (DA-ANN) to identify the intention of pedestrians. The accuracy of the model reached 95%.

In references [19,20,21,28], the intention parameter set used by the authors only includes the distance between pedestrian and zebra crossing, or the distance between the vehicle and zebra crossing, ignoring the influence of pedestrian speed and vehicle speed on pedestrian crossing intention, which has a certain impact on recognition accuracy. In addition, in reference [22], the number of samples used by the author in model training is limited. As such, although the accuracy of model recognition is high, there may be overfitting problems. In reference [7], when the model is 0.3 s ahead of time to identify the pedestrian intention, the recognition accuracy is not high, reaching only 80%. Motivated by the analysis of the existing works in related literatures, the characteristic variables that affect pedestrian crossing intention are analyzed in this work, a more comprehensive intention parameter set is obtained, and the long short-term memory network with attention mechanism(AT-LSTM) algorithm is adopted to improve the recognition accuracy of the model. 

**Figure 1:**
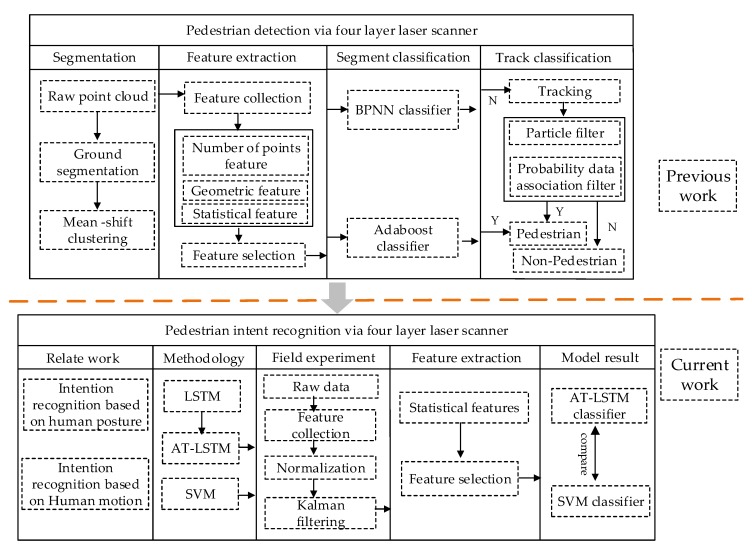
Pedestrian crossing intention recognition framework.

A mature vehicle-pedestrian intention recognition system must include pedestrian detection, tracking, and pedestrian intention recognition. Previous research [29] by the authors has provided a relevant analysis of pedestrian detection and tracking and achieved accurate detection and tracking of pedestrians. The recognition of pedestrian intention is the focus of this work on the basis of the previous research. As shown in Figure 1, this work can be divided into five parts. Section 1 presents the research background and related work which currently can be categorized as research that is based on posture language and research that is data-driven. Section 2 discusses research methodology, in which the AT-LSTM and support vector machine (SVM) algorithms are introduced along with algorithm parameter setting and model input variables. Section 3 details the parameters of pedestrians and vehicles, which are mainly collected by four-layer laser scanner and HD camera, followed by data normalization and filtering processing. Section 4 provides statistical analysis results of characteristic parameters. In this part, the variables closely related to pedestrian crossing intention are determined through statistical analysis. In Section 5 the results of recognition at 0 s and 0.6 s in advance using the two models are analyzed and the performance of the model with specific evaluation metrics is determined.

The main framework of this paper follows that of the previous research into the detection and tracking of pedestrians. The current work recognizes pedestrian crossing intention to form a complete system. The main framework is shown in Figure 1.

## 2. Method

### 2.1. Long Short-Term Memory Network (LSTM)

Long short-term memory network (LSTM) was first proposed by Hochreiter and Schmidhuber (1997) to solve the problem of gradient disappearance in recurrent neural networks. The key to this network is that the LSTM unit selectively adds or deletes some information through the gate structure, providing a mechanism to allow information to pass through selectively. The LSTM unit has three gate structures (input gate, forgetting gate, and output gate) to maintain and update the cell state. Here, $i_{t}$ $f_{t}$, $o_{t}$, and $C_{t}$ are used to represent the three gate structures and nerve cell states corresponding to t time. The details are provided as follows:

(1) The first step of LSTM is to delete some information in the nerve cells. Determined by the sigmoid layer of forgetting gate, its input is the data input $x_{t}$ of the current layer and the output $h_{t-1}$ of hidden layer from its upper layer, as shown in Equation (1):
  (1) $$f_{t}=\sigma \left(W_{f}\cdot \left[h_{t-1},x_{t}\right]+b_{f}\right)$$
  where σ represents sigmoid activation function and $W_{f}$ represents cyclic weight.
(2) The second step works to determine which new information should be stored in the cell state. It consists of two parts: (a) the input gate is determined by the sigmoid layer to be updated; (b) the new candidate value $\tilde{C_{t}}$ is created by the tanh layer and added to the nerve cells. This process is denoted in Equations (2) and (3):
  (2) $$i_{t}=\sigma \left(W_{t}\cdot \left[h_{t-1},x_{i}\right]+b_{i}\right)$$
  (3) $${\tilde{C}}_{t}=tanh\left(W_{c}\cdot \left[h_{t-1},x_{i}\right]+b_{c}\right)$$
  where $W_{t}$ represents cyclic weight and $b_{i}$ represents input bias.
(3) By calculating Equations (1)–(3), the state of the whole nerve cell is updated. Firstly, the original state of the nerve cell $C_{t-1}$ is multiplied by $f_{t}$ to delete the information that is useless and should be discarded, then $i_{t}\cdot \tilde{C_{t}}$ is added to determine the current update value of the state of the nerve cell, as illustrated in Equation (4):
  (4) $$C_{t}=f_{t}\cdot C_{t-1}+i_{t}\cdot {\tilde{C}}_{t}$$
(4) Finally, the information output is obtained through the output gate. Firstly, the sigmoid layer is used to determine which part of the information of the state of nerve cells will be output. Tanh is then employed to process the state of the nerve cells. Finally, the two parts of information are multiplied to determine the information to be output. This process is shown in Equation (5):
  (5) $$o_{t}=\sigma \left(W_{o}\cdot \left[h_{t-1},x_{t}\right]+b_{o}\right)$$
(5) The last output of the LSTM unit is $h_{t}$ and its formula is provided below:
  (6) $$h_{t}=o_{t}\cdot tanh\left(C_{t}\right)$$

### 2.2. Long Short-Term Memory Network Model with Attention Mechanism (AT-LSTM)

**Figure 2:**
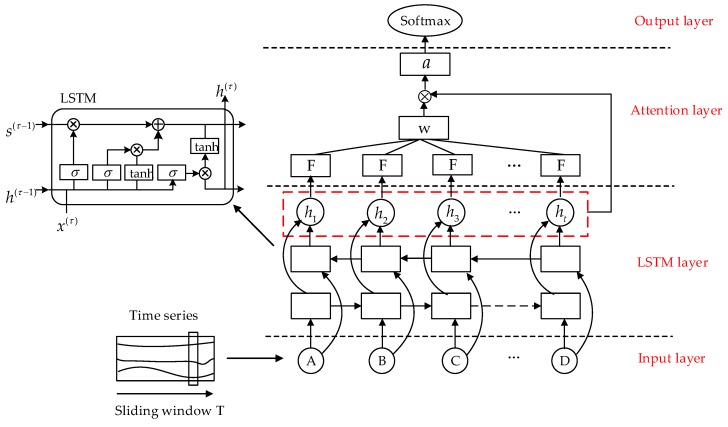
LSTM framework for integrating attention mechanism.

The attention mechanism works by imitating the human selective attention mechanism and operates by scanning data, focusing on data information. To obtain more information, increased attention is given to the details of the target, while other useless information is inhibited. Thus, the use of limited attention resources works to quickly screen out high-value data from a large amount of information to meet the pedestrian crossing intention discriminant of feature information processing efficiency and accuracy. In this work, the problem of pedestrian crossing intention recognition is regarded as a modeling and classification problem of time series. As both key features and redundant features will exist in the feature sequence extracted multiple times, applying attention mechanism will ensure greater weight is given to the key features in the modeling process, thus improving the efficiency and accuracy of the model prediction. Figure 2 illustrates how the attention mechanism is introduced into the LSTM framework for pedestrian crossing intention recognition.

In the LSTM framework, a learning function F is introduced (here, the learning function is realized through the fully connected layer). The learning function is used to calculate the weight $W_{t}$ of the output vector $h_{t}$ of the LSTM layer, and the final feature representation vector a is obtained by weighting. Finally, the recognition result of the pedestrian crossing intention is transmitted through the softmax layer. The calculation formula is shown in Equation (7):

(7) $$e_{t}=F\left(h_{t}\right)$$

where $h_{t}$ represents the output of the LSTM layer at time t. The calculation formula of weight $W_{t}$ is:

(8) $$W_{t}=\frac{\exp\left(e_{t}\right)}{\sum_{i=1}^{n}\exp\left(e_{i}\right)}$$

The formula is then weighted to obtain feature representation vector a, and its expression is shown in Equation (9):

(9) $$a=\overset{n}{\underset{t=1}{\sum }}\exp\left(W_{t}\;h_{t}\right)$$

### 2.3. AT-LSTM Model Input and Parameter Setting

Pedestrian crossing intention recognition can be regarded as a time series modeling and prediction problem. In this paper, intention association features are extracted through the continuous data flow of time series before the pedestrian and vehicle cross the street, then AT-LSTM is adopted for classification of pedestrian crossing intention. As detailed in the parameter analysis results, model input includes the seven characteristic parameters of vehicle speed, the distance between vehicle and zebra crossing, pedestrian speed, distance between pedestrian and zebra crossing, time to collision (TTC), age, and gender.

The time series of each characteristic parameter T-0 s is expressed as a characteristic vector. Additionally, $S_{veh}$, $Dis_{veh}$, $S_{ped}$, $Dis_{ped}$, TTC, Age, and Gen are used to represent the above parameters, respectively. Seven eigenvectors form the eigenmatrix. The input of the model is $M^{T}=\left[S_{veh}^{T},Dis_{veh}^{T},S_{ped}^{T},Dis_{ped}^{T},TTC^{T},Age^{T},Gen^{T}\right]$, where T is the time series length of 0-T s. When the model is identified 0.6 s in advance, the input of the model is $M^{n}=\left[S_{veh}^{n},Dis_{veh}^{n},S_{ped}^{n},Dis_{ped}^{n},TTC^{n},Age^{n},Gen^{n}\right]$, where n is the length of 0.6-T s time series.

The parameter setting for the AT-LSTM algorithm is determined by the expert experience method, that is, after ensuring the correctness of data and network, the default super parameter setting is employed (a learning rate of 0.1, dropout rate of 0.5, the number of hidden units of 100, and the max epochs of 50), the change of loss is observed, the range of each super parameter is preliminarily determined, then parameters are adjusted. For each super parameter, only one parameter is adjusted each time and the loss change is observed. The AT-LSTM network is composed of four layers of the stack, the dropout rate is 0.4, the number of hidden units per layer is 128, and the activation function of a fully connected layer is ReLU, and the max epochs is 80. Adam optimizer is adopted in the network, with a learning rate of 0.01 and an attenuation of 0.9.

### 2.4. Support Vector Machine (SVM) Theory and Feature Set Parameter Input

The support vector machine was first proposed by Cortes and Vapnik in 1995 and provides significant advantages in the recognition of small samples, non-linear, and high-dimensional patterns. The SVM method has strong generalization and has been widely used in the field of pattern recognition.

Before using SVM for training, the data to be trained and the data to be tested need to be expressed in a certain format. The data representation format used in this paper is as follows: $m^{T}=\left[S_{veh}^{T},Dis_{veh}^{T},S_{ped}^{T},Dis_{ped}^{T},TTC^{T},Age^{T},Gen^{T},labels\right]$, where T is the time series length of 0-T s. When the model is identified 0.6 s in advance, the input of the model is $m^{n}=\left[S_{veh}^{n},Dis_{veh}^{n},S_{ped}^{n},Dis_{ped}^{n},TTC^{n},Age^{n},Gen^{n},labels\right]$, where n is the length of 0.6-T s time series.

### 2.5. Kernel Function Selection and Sarameter Optimization

Kernel function has a direct impact on the training classification accuracy and test recognition rate of the SVM model. At present, the selection of kernel functions is still the focus of a large number of researchers. Each kernel function has its own advantages and disadvantages. The characteristics of each kernel function are different. The characteristics of the SVM model built by different kernel functions are also different. Common kernel functions include the following: linear kernel, polynomial kernel, radial basis function kernel, and sigmoid tanh. By comparing the classification accuracy of different kernel functions, the radial basis function kernel is selected in this paper.

According to the formula of the kernel function, we can know that the parameter C and the kernel function parameter σ have a large influence on the performance of SVM. As the parameters can be searched in a wide range, this paper employs a genetic algorithm to optimize the parameters of the kernel function. In the case of five folds cross-validation, the optimal parameters C = 89.08, σ = 4.84 after optimization by genetic algorithm.

## 3. Experimental

### 3.1. Experimental Site

**Figure 3:**
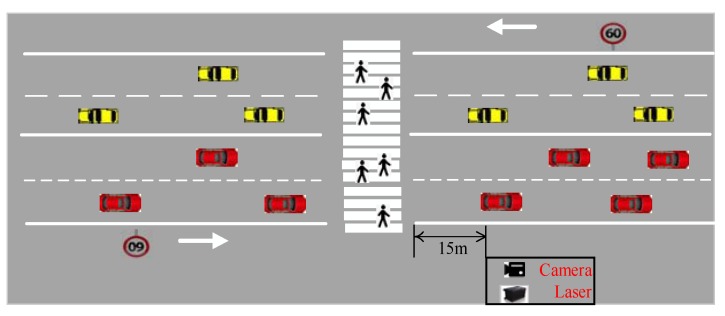

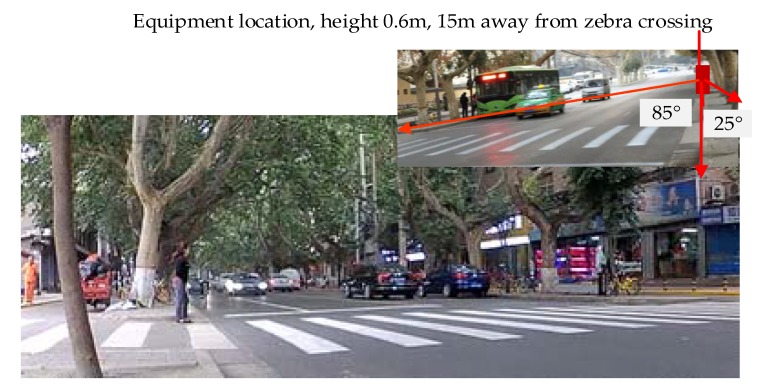
Photographs of the experimental section.

The zebra crossing selected in this paper is located at the west gate of Chang’an University and crosses Wenyi South Road. The width of the zebra crossing is 12 m, and it is located in a straight section of the two-way road with four lanes with a small and negligible slope. There is no signal light control at the site, a double yellow line in the middle of the road, and no refuge island, green belt, or monitor capturing device above the road. Approximately 30 m away from the zebra crossing there are signs for pedestrians and schools. The speed limit of this section is 60 km/h and the speed limit is set 100 m away from the zebra crossing. A sketch of the experimental section is provided in Figure 3. The traffic flow of the zebra crossing is mainly composed of taxis and private cars, accounting for more than 95% of the traffic, and the remaining traffic is predominantly buses and minivans. Traffic flow is about 600 veh/h in rush hours and 400 veh/h in non-rush hours.

### 3.2. Experimental Equipment

**Figure 4:**
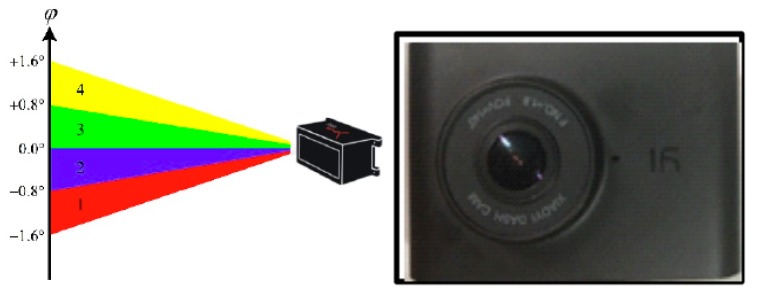
Parameter acquisition equipment.

The main equipment used in the experiment was a laser scanner and mini HD monitor, as shown in Figure 4. The laser scanner model was an IBEO LUX 4L-4 (IBEO Automotive Systems GmbH, Hamburg, DE, Deutschland) with a scanning frequency of 12.5 Hz, a detectable range of 0.3 m–200 m, and a vertical viewing angle of 3.2° FOV. The Ilv-Premium software associated with a laser scanner can display the target type (car, bus, pedestrians), position, and speed in real-time, and all data can be stored and replayed. The video resolution of the mini HD monitor was 1920 ∗ 1080. The left half of the image in Figure 4 shows the laser scanner; the right half of the image is the HD monitor. The experimental equipment was placed on the left side of the road, 15 m away from the zebra crossing, equipment 0.6 m away from the ground and the laser scanner could effectively cover both sides of the road, as shown in Figure 3. In this paper, as the laser scanner could not see whether the pedestrian had reached the curb, an HD monitor and laser scanner time synchronization was used to determine the position of the pedestrian at different times. Secondly, a camera was also used to determine the gender and observation age of pedestrians. All equipment was placed in relatively concealed locations to avoid interference with vehicles and pedestrians. In order to protect personal privacy, the recorded video data was used only for scientific research.

### 3.3. Data Collection and Analysis

All data observation and collection experiments were conducted on sunny days to avoid the influence of the weather. From 2016 to 2018, several data collections were carried out in May each year, with each collection period lasting 1 h. Collection periods included morning and evening rush hours, and more than 90 h of experimental data were obtained in total. 

#### Event-Labeling methodology

The event labeling guidelines proposed in this paper carry out identification the instant that a pedestrian starts or finishes an activity. In this work, pedestrian crossing intention is divided into three categories: walking-stopping, walking-walking, and stopping-starting. Specifically, a walking-stopping activity is defined as the action when the pedestrian gets closer to the zebra crossing and the speed of the pedestrian decreases and finally becomes 0 km/h, stopping in front of the zebra crossing. Walking-walking activity is defined as the action when pedestrians cross the street directly without any stop and the speed is always greater than 0 km/h in the process. A stopping-starting activity is defined as the action from the initial standstill in front of the zebra crossing to the beginning of a pedestrian’s motivation to cross the street. This criterion was adopted because these transitions are easily labeled by human experts, thus enabling the creation of reliable ground truths.

**Figure 5:**
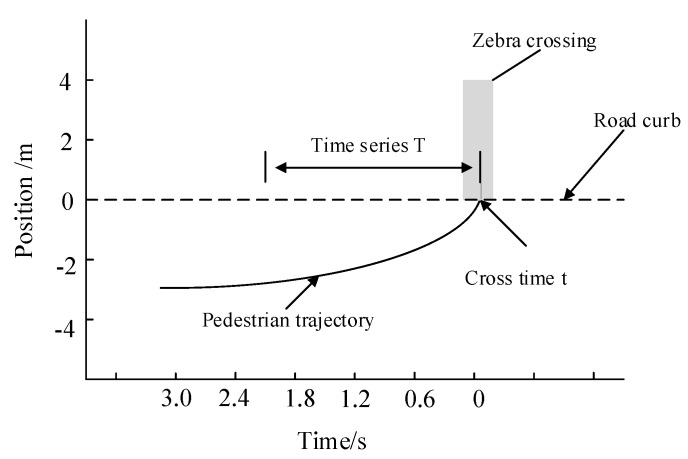
Time series of pedestrian crossing.

To complete more accurate modeling of pedestrian crossing intention, it was also necessary to extract information of surrounding vehicles and the movement information of the pedestrians themselves. The extracted data included the distance between pedestrians and zebra crossing, pedestrian speed, vehicle speed, the distance between vehicles and zebra crossing, time to collision (TTC), and the age and gender of pedestrians. The parameter of crossing intention was extracted according to the following steps:

(1) Valid samples of three types of crossing (walking-stopping, walking-walking, and stopping-walking) are obtained.
(2) The instantaneous moment t when the pedestrians cross the curb or stop at the curb is determined.
(3) The instantaneous time t is taken as the starting point, then the time series parameters of pedestrian T s before crossing the curb is reverse extracted (the parameters are described above), as shown in Figure 5.
(4) The instantaneous time t is taken as the starting point, then the time series parameters of vehicle in 0-T s are extracted in reverse.

Using the above process, a total of 1980 effective samples were extracted, which included 680 walking-stopping groups, 658 walking-walking groups, and 642 stopping-starting groups. The process of model recognition is thus essentially a three-class classification problem.

Detailed definitions of some parameters are provided as follows:

Distance between pedestrian and zebra crossing (m)-$Dis_{ped}$: refers to the arithmetic square root of the sum of the square of the longitudinal distance ($dis_{lon}$) between pedestrian and curb and the square of the transverse distance($dis_{tran}$):

(10) $$Dis_{ped}=\sqrt{dis_{lon}{}^{2}+dis_{tran}{}^{2}}$$

Distance between vehicle and zebra crossing (m)-$Dis_{veh}$: refers to the vertical distance between the current location of the vehicle and the location of zebra crossing.

TTC (s): refers to the ratio of the distance between the vehicle and the zebra crossing to the vehicle speed:

(11) $$TTC=\frac{Dis_{veh}}{S_{veh}}$$

In this paper, the vehicle speed and the distance between vehicles and zebra crossing are much higher than other parameters. Inputting data with different value range into the recurrent neural network creates a problem because while the network may adapt the data with a different value range, it will become more difficult to learn. Therefore, all parameters are normalized to improve the learning efficiency and recognition accuracy of the model. Meanwhile, due accuracy limitations of the acquisition instrument, the parameters selected in the feature set have a certain step in the data obtained in the actual test process, which may weaken the correlation between the data. The most important task in the process of identifying the intention to cross the street is to mine the relevant characteristics of the data from the pedestrian crossing data and the surrounding vehicle data. To eliminate the partial step of the original collected data and ensure the pedestrian crossing intention recognition model maintains high accuracy, the intention parameter set is filtered. For most problems, the Kalman filter is the most effective and efficient method and is selected to filter the intention parameter set in this work.

## 4. Feature Parameter Analysis Results

### 4.1. Age and Gender

There are many parameters that influence pedestrian crossing intention. From the perspective of psychology, Pei et al. [30] and Guo et al. [31] found that pedestrian crossing decision making is most correlated with age. Young and middle-aged pedestrians are relatively radical when crossing the street. When the external environment allows, the probability of inducing these pedestrians to cross the street is relatively high. However, as the elderly are less physically capable, they are more cautious when crossing the street. They often avoid vehicles when crossing, and the probability of inducing the elderly to cross the street is relatively low when the external conditions allow. To improve the training accuracy of the model, the pedestrian’s age was divided. according to natural observation, using the classification method mentioned in the references which define 18–30 as a youth, 30–59 as middle age, and >60 as old age [32,33,34].

### 4.2. Distance between Vehicle and Zebra Crossing

**Figure 6:**
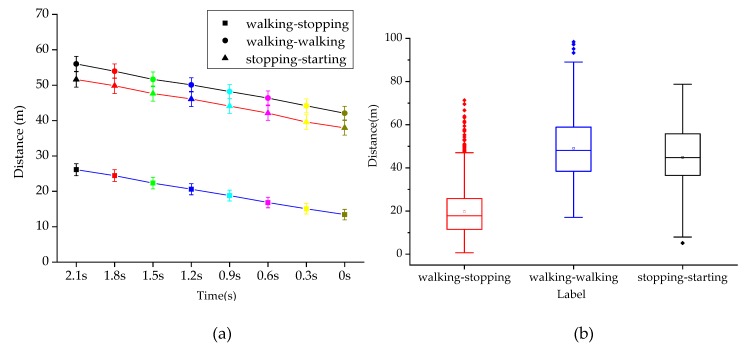
Distance between vehicles and zebra crossings under different crossing intentions. (**a**) The distance between vehicles and zebra crossings at different times and with different intentions. (**b**) Mean distance between vehicles and zebra at crossings under different intentions.

As shown in Figure 6a, the error bar diagram of T-0 s before crossing the street under three pedestrian intentions is drawn at the interval of 0.3 s, respectively. The main effect analysis shows that pedestrian crossing intention is significantly related to the distance between the vehicle and zebra crossing (*p* < 0.001), and the difference of time before crossing also significantly affects the distance between the vehicle and zebra crossing (*p* < 0.001). It is also observed that there is no interaction effect between the time of the street crossing in advance and the intention of the street crossing (*p* > 0.05). Therefore, the interaction of the two factors has no significant effect on the distance between vehicles and zebra crossing. The three polylines in the figure have no intersection, which also indicates that there is no interaction effect, consistent with the results in Table 1.

As shown in Figure 6b, when the intention of pedestrians is walking-stopping, the mean distance between vehicles and zebra crossing is 19.44 m; when the intention of pedestrians is walking-walking, the mean distance between vehicles and zebra crossing is 49.28 m; when the intention of pedestrians is stopping-walking, the mean distance between vehicles and zebra crossing is 45.13 m. The one-way analysis of variance (ANOVA) test shows that the distance between vehicles and zebra crossings can significantly affect pedestrian crossing intentions (F (2,15840) = 2247.65, *p* < 0.001). The post-hoc comparisons are provided in Table 2. It can be seen that the mean distance between vehicles and zebra crossing under walking-stopping and walking-walking, the mean distance between vehicles and zebra crossing under walking-walking and stopping-starting, and the mean distance between vehicles and zebra crossing under walking-stopping and stopping-starting are all significantly different (*p* < 0.001).

### 4.3. Vehicle Speed

**Figure 7:**
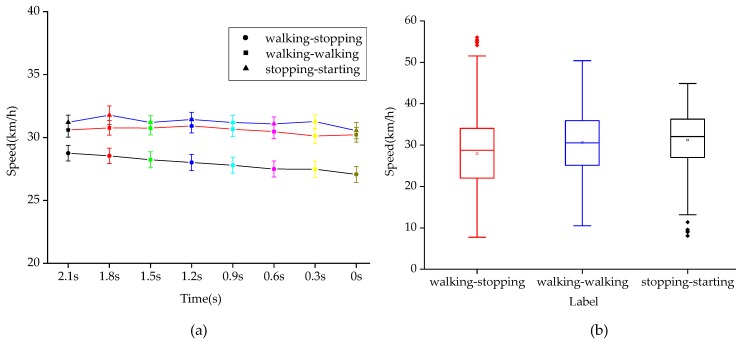
Vehicle speed diagram under different crossing intentions. (**a**) Vehicle speed at different times with different intentions. (**b**) Mean vehicle speed under different intentions.

As shown in Figure 7a, the error bar graph of vehicle speed in T-0 s before crossing under three pedestrian intentions is drawn at 0.3 s intervals. The main effect analysis shows that vehicle speed demonstrates significant difference under different crossing intentions (*p* < 0.001), while the influence of time change before pedestrian crossing on vehicle speed is not significant (*p* > 0.05). In addition, there is no interaction effect between the advance time of crossing the street and the intention of pedestrians (*p* > 0.05). It can be seen that the interaction of the two factors has no significant effect on vehicle speed. The absence of any intersection between the three polylines in the figure also indicates that there is no interaction effect, which is consistent with the results in Table 3.

As shown in Figure 7b, when the intention of pedestrians is walking-stopping, the mean speed of vehicles is 29.94 km/h; when the intention of pedestrians is walking-walking, the mean speed of vehicles is 30.61 km/h; when stopping-walking is the intention of pedestrians, the mean speed of vehicles is 31.21 km/h. The one-way ANOVA test shows that the mean vehicle speed under different intention labels displays a significant difference (F (2,15840) = 83.69, *p* < 0.001). The post-hoc comparisons are shown in Table 4. It can be seen that the mean vehicle speed under walking-stopping and walking-walking is significantly different (*p* < 0.001); the mean vehicle speed under walking-walking and stopping-starting have no significantly different (*p* = 0.15 > 0,05); the mean vehicle speed under walking-stopping and stopping-starting are significantly different (*p* < 0.001).

### 4.4. Time to Collision (TTC)

**Figure 8:**
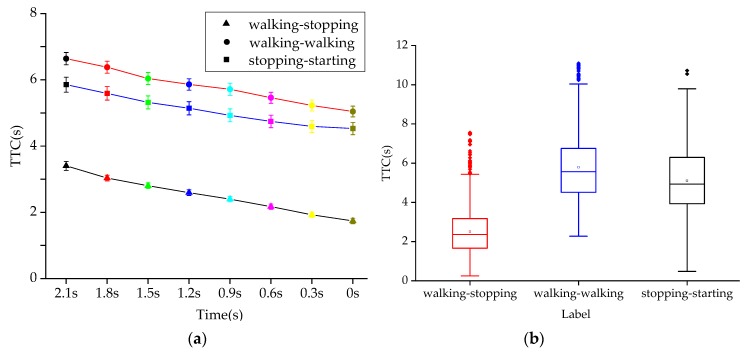
TTC diagram under different crossing intentions. (**a**) TTC at different times and with different intentions (**b**) TTC under different intentions.

As shown in Figure 8a, the error bar graph of TTC in T-0 s before crossing under three pedestrian intentions is drawn at 0.3 s intervals. The main effect analysis shows that there are significant differences in TTC under different crossing intentions (*p* < 0.001) and TTC under different crossing moments (*p* < 0.001), as shown in Table 5. The table also illustrates that the interaction effect between different intentions and times is not significant (*p* > 0.05), which is further demonstrated by the absence of any intersection of the three polylines in Figure 8a. It can be seen that the interaction of the two factors has no significant effect on the TTC.

As shown in Figure 8b, when the intention of pedestrians is walking-stopping, the mean TTC is 2.51 s; when the intention of pedestrians is walking-walking, the mean TTC is 5.79 s; when the intention of pedestrians is stopping-walking, the mean TTC is 5.11 s. The one way ANOVA test shows that the mean TTC value under different intentions is significantly different (F (2,15840) = 1719.60, *p* < 0.001). The post-hoc comparisons are shown in Table 6 and post-hoc comparisons are provided in Table 2. It can be seen that the mean TTC under walking-stopping and walking-walking, the mean TTC under walking-walking and stopping-starting, and the mean TTC under walking-stopping and stopping-starting are all significantly different (*p* < 0.001).

### 4.5. Pedestrian Speed

**Figure 9:**
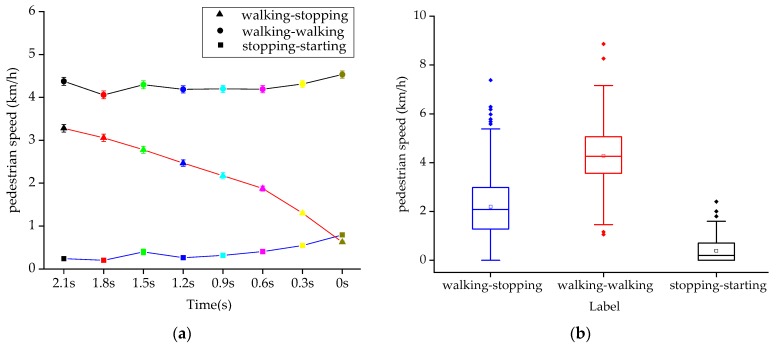
Pedestrian speed diagram under different crossing intentions. (**a**) Pedestrian speed at different times and with different intentions. (**b**) Mean pedestrian speed under different intentions.

As shown in Figure 9a, the error bar graph of pedestrian speed in T-0 s before crossing under different pedestrian intentions is drawn at 0.3 s intervals. The main effect analysis shows that there is a significant influence on pedestrian speed under different crossing intentions and pedestrian speed under different crossing moments (*p* < 0.001), as shown in Table 7. The interaction effect between different intentions and different moments is significant (*p* < 0.001), and the three polylines in Figure 9a have intersection points, indicating the existence of interaction effects. Therefore, the interaction of two factors can significantly affect the pedestrian speed.

As illustrated in Figure 9b, when the intention of pedestrians is walking-stopping, the mean pedestrian speed is 2.22 km/h; when the intention of pedestrians is walking-walking, the mean pedestrian speed is 4.27 km/h; when the intention of pedestrians is stopping-walking, the mean pedestrian speed is 0.39 km/h. The one way ANOVA test shows that the mean pedestrian speed value under different intentions is significantly different (F (2,15840) = 2274.09, *p* < 0.001). The post-hoc comparisons are shown in Table 8. 

### 4.6. Distance between Pedestrians and Zebra Crossings

**Figure 10:**
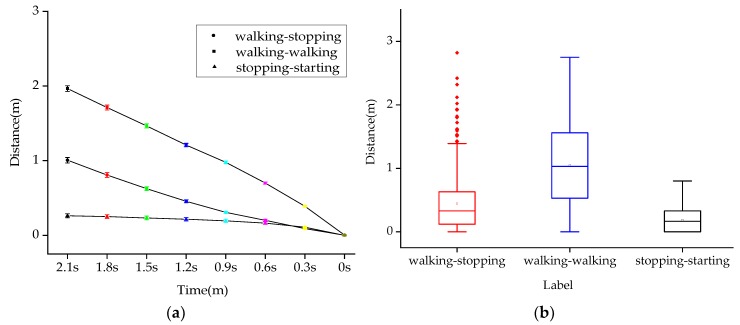
Distance between pedestrians and zebra crossings under different crossing intentions. (**a**) Distance between pedestrians and zebra crossings at different times and with different intentions. (**b**) Mean distance between pedestrians and zebra crossings under different crossing intentions.

As shown in Figure 10a, the error bar graph of the distance between pedestrians and zebra crossings in T-0 s before crossing under different pedestrian intentions is drawn at 0.3 s intervals. The main effect analysis shows that there are significant differences in the distance between pedestrians and zebra crossings under different crossing intentions and the distance between pedestrians and zebra crossings under different crossing moments (*p* < 0.001), as shown in Table 9. The interaction effect between different intentions and different moments is significant (*p* < 0.001). It can be seen that the interaction of two factors can significantly affect Distance between pedestrians and zebra crossings. The three polylines in Figure 10a have intersection points, indicating the existence of interaction effects.

As shown in Figure 10b, when the intention of pedestrians is walking-stopping, the mean distance between pedestrians and zebra crossings is 0.44 m; when the intention of pedestrians is walking-walking, the mean distance between pedestrians and zebra crossings is 1.05 m; when the intention of pedestrians is stopping-walking, the mean distance between pedestrians and zebra crossings is 0.18 m. The one way ANOVA test shows that the mean pedestrian speed value under different intentions is significantly different (F(2,15840) = 2018.46, *p* < 0.001). Post-hoc comparisons are shown in Table 10. It can be seen that the mean distance between pedestrians and zebra crossings under walking-stopping and walking-walking, the mean p distance between pedestrians and zebra crossings under walking-walking and stopping-starting, and the mean distance between pedestrians and zebra crossings under walking-stopping and stopping-starting are all significantly different (*p* < 0.001).

## 5. Model Analysis Results

In this experiment, a total of 1980 effective samples were collected, 80% of which are used as training sets and the remaining 25% as test sets for model training, as shown in Table 11. The AT-LSTM algorithm is predominantly used for model training, and the SVM algorithm was used for model accuracy comparison. An analysis of the accuracy of the model to recognize the intention of crossing 0 s in advance and 0.6 s in advance is the primary focus of this work.

### 5.1. Model Recognition Results 0 s in Advance

Recognition results based on the AT-LSTM network model and SVM model for when the input feature time series is T-0 s, that is, the intention of pedestrians is not recognized in advance, are shown in Table 12. It can be seen from the recognition accuracy that the network model based on AT-LSTM has a higher recognition accuracy than the SVM model. The pedestrian intention recognition accuracy of the AT-LSTM model is 96.15%, while the pedestrian intention recognition accuracy of the SVM model is 90.08%. Therefore, it is more advantageous to identify pedestrian intention based on the AT-LSTM model. In addition, the model needs to have the ability of real-time computing. The computing time of AT-LSTM and SVM models is 0.0049s and 0.0036s respectively through tic and toc functions. We trained the model with a CPU of core i5-7th. The training time of model AT-LSTM and SVM model is 3 min 42 s and 4 min 7 s respectively.

**Figure 11:**
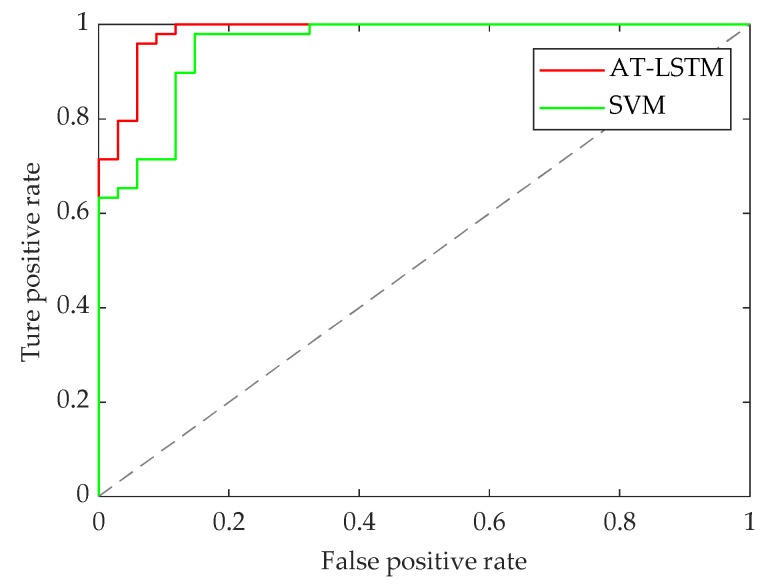
ROC curve of model identification 0 s in advance.

The following Figure 11 shows the receiver operating characteristic (ROC) curves of the two recognition models, in which the curves deviate far from the 45° oblique line. The results illustrate that both models have good recognition performance.

**Figure 12:**
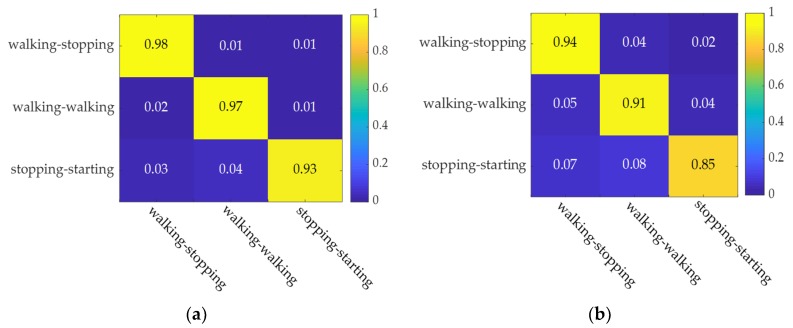
Confusion matrix for model identification 0 s in advance. (**a**) AT-LSTM model. (**b**) SVM model.

Figure 12 shows the confusion matrix based on the AT-LSTM model and SVM model. The figure illustrates the model recognition accuracy under the three types of labels, in which the two models display good recognition accuracy. 

### 5.2. Model Performance Evaluation 0 s in Advance

Precision recall rate and F1 scores are used to further evaluate the classification performance of the models, with results provided in Table 13. It can be seen from Table 13 that the classification performance of the AT-LSTM model is significantly better than that of the SVM model in terms of the precision, recall rate, and F1 scores of the model.

### 5.3. Model Recognition Results 0.6 s in Advance

Recognition results based on the AT-LSTM network model and SVM model are shown in Table 14 for when the length of the input characteristic time series is T-0.6 s, that is, the pedestrian crossing intention is recognized 0.6 s in advance. According to the results, the recognition accuracy of the AT-LSTM network model is higher than that of the SVM model. The accuracy of pedestrian intention recognition of the AT-LSTM model is 90.68%, and that of the SVM model is 85.83%. It can also be observed that when crossing intention occurs 0.6 s in advance, the pedestrian crossing intention recognition also has high accuracy. The accuracy of pedestrian crossing intention recognition 0.6 s in advance is generally lower than that of 0 s in advance. In addition, the computing time of AT-LSTM and SVM models is 0.0023 s and 0.0054 s respectively through tic and toc functions. The training time of model AT-LSTM and SVM model is 2 min 36 s and 2 min 52 s respectively.

**Figure 13:**
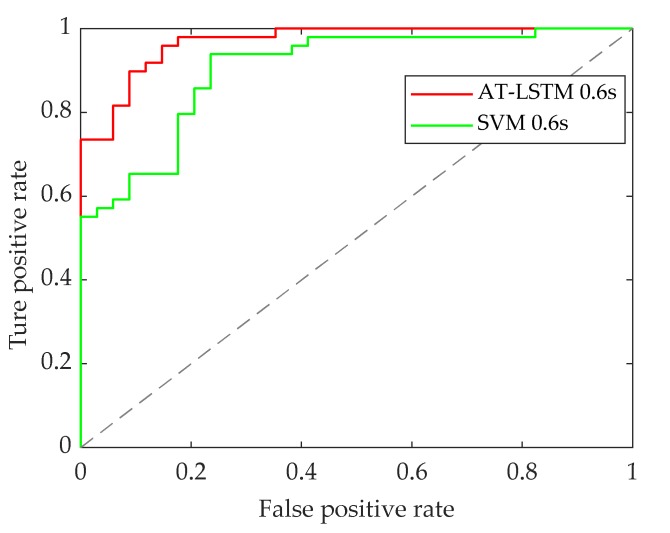
ROC curve of model identification 0.6 s in advance.

The following Figure 13 shows the ROC curves of the two recognition models. It can be seen that when the pedestrian crossing intention is recognized 0.6 s in advance, the two curves display relative deviation from the 45° diagonal, illustrating that both models have good recognition performance.

**Figure 14:**
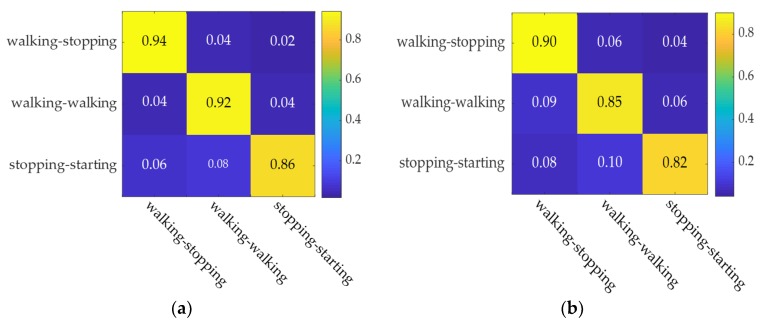
Confusion matrix for model identification 0.6 s in advance. (**a**) AT-LSTM model. (**b**) SVM model.

Figure 14 respectively show the confusion matrix based on the AT-LSTM model and SVM model when pedestrian crossing intention recognition occurs 0.6 s in advance. 

The figures illustrate model recognition accuracy under the three types of labels in which the two models display good recognition accuracy. It is worth noting that the model recognition accuracy is generally lower when pedestrian crossing intention recognition occurs 0.6 s before crossing compared to when pedestrian crossing intention recognition occurs 0 s before crossing.

### 5.4. Model Performance Evaluation 0.6 s in Advance

The performance of the two models when the pedestrian intention is recognized 0.6 s in advance is evaluated according to precision, recall rate, and F1 scores of the models as listed in Table 15. It can be seen from these results that the recognition performance of the AT-LSTM model is still superior to the SVM model. 

## 6. Conclusions

In this paper, the motion parameters of pedestrians and vehicles were collected by a four-layer laser scanner and 1980 groups of effective samples were selected. The statistical method was then employed to test the significance of the selected data, and a more comprehensive set of characteristic parameters that can reflect the intention of pedestrians to cross the street was obtained. It is determined that TTC has a significant impact on pedestrian crossing intention, which is consistent with the results of the literature [35]. In addition, as consistent with the research results in the literature [19,20,21,28], the distance between pedestrians and zebra crossing and the distance between vehicles and zebra crossing have a significant impact on pedestrian crossing intention. Contrasting from the above studies, pedestrian crossing speed and the vehicle speed is also determined to have a significant impact on pedestrian crossing intention, which enriches the pedestrian crossing intention parameter set and lays a foundation for more scholars to carry out real-time online pedestrian crossing intention research.

Taking the feature parameter set as the input of the AT-LSTM algorithm, a pedestrian crossing intention model with high recognition accuracy was trained and compared with the traditional SVM algorithm. Results illustrated that while the two models have high recognition accuracy, the AT-LSTM model provides more advantages for pedestrian crossing intention recognition, reaching 96.15%. This result is 6.07% higher than the SVM model. In addition, when the proposed model recognized the intention of pedestrians crossing the street 0.6 s in advance, it was still able to complete accurate recognition, with the recognition accuracy reaching 90.68%, 4.85 % higher than the SVM model. The AT-LSTM model proposed in this paper has a high accuracy of intention recognition, which is of practical significance for future fully automated driving vehicles to effectively avoid human vehicle conflict and improve driving efficiency on urban roads.

## Figures and Tables

**Table 1:** Main effect test table.

Source	df	F	Sig.
Label (walking-smiddleping, walking-walking, smiddleping-starting)	2	2690.34	00
Time (0-T s)	7	63.86	00
Label * Time	14	035	1.00
Total number	15,840		

**Table 2:** Post-hoc comparison table.

Label		Std Error	Sig.
walking-smiddleping	walking-walking	15	00
	smiddleping-starting	26	00
walking-walking	walking-smiddleping	36	00
	smiddleping-starting	19	00
smiddleping-starting	walking-smiddleping	15	00
	walking-walking	26	00

**Table 3:** Main effect test table.

Source	df	F	Sig.
Label (walking-smiddleping, walking-walking, smiddleping-starting)	2	92.46	00
Time (0-T s)	7	1.51	16
Label * Time	14	63	0.84
Total number	15,840		

**Table 4:** Post-hoc comparison table.

Label		Std Error	Sig.
walking-smiddleping	walking-walking	16	00
	smiddleping-starting	22	00
walking-walking	walking-smiddleping	17	00
	smiddleping-starting	20	15
smiddleping-starting	walking-smiddleping	21	00
	walking-walking	23	15

**Table 5:** Main effect test table.

Source	df	F	Sig.
Label (walking-smiddleping, walking-walking, smiddleping-starting)	2	1973.26	00
Time (0-T s)	7	40.35	00
Label * Time	14	14	0.89
Total number	15,840		

**Table 6:** Post-hoc comparison table.

Label		Std Error	Sig.
walking-smiddleping	walking-walking	03	00
	smiddleping-starting	04	00
walking-walking	walking-smiddleping	04	00
	smiddleping-starting	05	00
smiddleping-starting	walking-smiddleping	03	00
	walking-walking	04	00

**Table 7:** Main effect test table.

Source	df	F	Sig.
Label (walking-smiddleping, walking-walking, smiddleping-starting)	2	2985.39	00
Time (0-T s)	7	21.08	00
Label * Time	14	60.86	00
Total number	15,840		

**Table 8:** Post-hoc comparison table.

Label		Std Error	Sig.
walking-smiddleping	walking-walking	03	00
	smiddleping-starting	04	00
walking-walking	walking-smiddleping	03	00
	smiddleping-starting	04	00
smiddleping-starting	walking-smiddleping	04	00
	walking-walking	04	00

**Table 9:** Main effect test table.

Source	df	F	Sig.
Label (walking-smiddleping, walking-walking, smiddleping-starting)	2	2263.66	00
Time (0-T s)	8	570.24	00
Label * Time	14	133.48	00
Total number	15,840		

**Table 10:** Post-hoc comparison table.

Label		Std Error	Sig.
walking-smiddleping	walking-walking	01	00
	smiddleping-starting	02	00
walking-walking	walking-smiddleping	01	00
	smiddleping-starting	02	00
smiddleping-starting	walking-smiddleping	02	00
	walking-walking	01	00

**Table 11:** Number of intention samples.

Label	Train Sample	Test Sample
walking-smiddleping	510	170
walking-walking	494	164
smiddleping-walking	482	160

**Table 12:** Model recognition accuracy of 0 s in advance.

Total Number	SVM Model	AT-LSTM Model
1980	90.08%	96.15%

**Table 13:** Model performance evaluation.

Model	Precision	Recall Rate	F1 Scores
AT-LSTM	95.43%	98.24%	96.81%
SVM	89.89%	94.12%	91.96%

**Table 14:** Model recognition accuracy for 0.6 s in advance.

Total Number	SVM Model	AT-LSTM Model
1980	85.83%	90.68%

**Table 15:** Model performance evaluation.

Model	Precision	Recall Rate	F1 Scores
AT-LSTM	90.34%	93.53%	91.91%
SVM	85.08%	90.58%	87.74%
